# Meta-omic profiling reveals ubiquity of genes encoding for the nitrogen-rich biopolymer cyanophycin in activated sludge microbiomes

**DOI:** 10.3389/fmicb.2023.1287491

**Published:** 2023-11-16

**Authors:** McKenna Farmer, Rashmi Rajasabhai, William Tarpeh, Keith Tyo, George Wells

**Affiliations:** ^1^Civil and Environmental Engineering, Northwestern University, Evanston, IL, United States; ^2^Chemical and Biological Engineering, Northwestern University, Evanston, IL, United States; ^3^Chemical Engineering, Stanford University, Stanford, CA, United States

**Keywords:** nitrogen recovery, microbial ecology, cyanophycin, activated sludge, phosphorus accumulating organisms

## Abstract

Recovering nitrogen (N) from municipal wastewater is a promising approach to prevent nutrient pollution, reduce energy use, and transition toward a circular N bioeconomy, but remains a technologically challenging endeavor. Existing N recovery techniques are optimized for high-strength, low-volume wastewater. Therefore, developing methods to concentrate dilute N from mainstream wastewater will bridge the gap between existing technologies and practical implementation. The N-rich biopolymer cyanophycin is a promising candidate for N bioconcentration due to its pH-tunable solubility characteristics and potential for high levels of accumulation. However, the cyanophycin synthesis pathway is poorly explored in engineered microbiomes. In this study, we analyzed over 3,700 publicly available metagenome assembled genomes (MAGs) and found that the cyanophycin synthesis gene *cphA* was ubiquitous across common activated sludge bacteria. We found that *cphA* was present in common phosphorus accumulating organisms (PAO) *Ca.* ‘Accumulibacter’ and *Tetrasphaera,* suggesting potential for simultaneous N and P bioconcentration in the same organisms. Using metatranscriptomic data, we confirmed the expression of *cphA* in lab-scale bioreactors enriched with PAO. Our findings suggest that cyanophycin synthesis is a ubiquitous metabolic activity in activated sludge microbiomes. The possibility of combined N and P bioconcentration could lower barriers to entry for N recovery, since P concentration by PAO is already a widespread biotechnology in municipal wastewater treatment. We anticipate this work to be a starting point for future evaluations of combined N and P bioaccumulation, with the ultimate goal of advancing widespread adoption of N recovery from municipal wastewater.

## 1. Introduction

Recovering nitrogen (N) from municipal wastewater is a promising method to circularize anthropogenic N use. Traditionally, fertilizer manufacturing and other industries synthetically fix N from the atmosphere through the energy intensive Haber Bosch process, which accounts for 1–2% of global energy use (Batstone et al., 2015). A significant portion of this reactive N is ultimately lost to municipal and industrial wastewater or agricultural drainage water. This reactive N harms aquatic environments and impacts public health (Galloway et al., 2008). Therefore, reducing N emissions to the environment is a key goal of wastewater treatment plants. Reactive N is typically removed from municipal wastewater through microbially driven redox reactions back to inert N_2_, which is dissipated back to the atmosphere. N recovery from wastewater is appealing because it can reduce N release to the environment while rerouting reactive N back to food or chemical production, promoting a transition from a linear to a circular anthropogenic N cycle and reducing reliance on Haber Bosch. However, existing N recovery techniques are optimized for high-strength, low-volume wastewater and are not feasible to apply to low-strength, high-volume mainstream municipal wastewater (Beckinghausen et al., 2020). Therefore, a partition-release-recovery (PRR) approach has been proposed to sequester nutrients from mainstream wastewater to a highly concentrated sidestream (Batstone et al., 2015). An efficient partition step to concentrate dilute N is an essential yet poorly explored element of this approach for N recovery.

A promising lead for N bioconcentration is cyanophycin, an intracellular biopolymer composed of amino acids aspartate and arginine. Cyanophycin is polymerized via cyanophycin synthetase, *cphA.* Cyanophycin can be synthesized *de novo* by cyanophycin synthetase, or existing cyanophycin can be used as a primer compound (Ziegler et al., 1998). Cyanophycin synthesis results in ATP hydrolysis to ADP (Berg et al., 2000). Cyanophycin is broken down into dipeptides by cyanophycinase, *cphB* (Richter et al., 1999; Supplementary Figure S1). Cyanophycin dipeptides may be hydrolyzed by isoaspartyl peptidase *iaaA*, though other enzymes can perform this function (Sharon et al., 2023). Cyanophycin was originally characterized in cyanobacteria and has also been studied in a limited number of non-phototrophic bacteria (Füser and Steinbüchel, 2007). In phototrophic and diazotrophic cyanobacteria, cyanophycin is most likely used as a nitrogen storage compound under alternating light conditions, during periods where nitrogen is in excess, and during periods where sulfate and phosphate are limited (Flores et al., 2019). In non-phototrophic bacteria, cyanophycin synthesis, prevalence, and function is poorly understood. It is not known if these selective pressures for cyanophycin synthesis are generalizable to other taxa, particularly to as-yet-uncultivated heterotrophs that are prevalent in wastewater treatment bioreactors. Studies on axenic *Acinetobacter* cultures have found that cyanophycin accumulation occurs under phosphate or sulfate limitation, as well as in the presence of ammonium or arginine in excess of what is needed for growth (Elbahloul et al., 2005).

In isolate cultures, cyanophycin can reach up to 40% of cell dry weight (Elbahloul et al., 2005). Cyanophycin granules can be selectively by manipulating pH, enabling straightforward purification (Füser and Steinbüchel, 2005). Given the attractive solubility properties, industrial biotechnology research has focused on maximizing cyanophycin production in recombinant bacteria, yeasts, and transgenic plants (Nausch et al., 2016; Du et al., 2019). Cyanophycin has been explored as a starter compound for a variety of downstream applications, including animal feedstock supplementation (Nausch et al., 2020), polyelectrolyte multilayer production for biomedical applications (Uddin et al., 2020), and feedstock for biodegradable plastic production (Neumann et al., 2005).

Cyanophycin production in mixed microbial communities, particularly in wastewater bioprocesses, has not been well-documented to date. Recent work has shown that cyanophycin can be produced unintentionally in activated sludge (Zou et al., 2022). Other meta-omic studies have incidentally identified cyanophycin synthetase genes in microbes commonly found in activated sludge (Füser and Steinbüchel, 2007; Singleton et al., 2022) but have not systematically searched for the cyanophycin pathway in wastewater bioprocesses. Cyanophycin accumulation could add immense value to existing biological nutrient removal practices, namely the enhanced biological phosphorus removal (EBPR) process. EBPR processes enrich phosphorus accumulating organisms (PAO), heterotrophs that release and uptake P under alternating redox conditions and substrate availability. Existing EBPR processes already use the PRR approach for P recovery, where P-rich biomass is bioconcentrated and physically separated from the dilute liquid stream. Therefore, integrating cyanophycin accumulation with existing P removal practices offers a lower barrier to entry for N recovery.

To better understand the potential role of cyanophycin as an N-rich biopolymer for the PRR approach, we assessed over 3,700 publicly available metagenome assembled genomes (MAGs) to understand the prevalence of the cyanophycin biosynthetic pathway in activated sludge microbiomes. We also curated MAGs and isolate genomes of key functional groups known to contribute to N cycling and P accumulation to determine their capability for cyanophycin accumulation. Finally, we analyzed gene expression data of known PAO to understand whether PAO could utilize cyanophycin synthesis genes. We found that genes enabling cyanophycin accumulation were ubiquitous amongst activated sludge communities, which may be leveraged in the future to partition and recover N as cyanophycin.

## 2. Materials and methods

### 2.1. Large metagenome-assembled genome datasets

Wastewater bioprocess MAGs were obtained from two primary sources that used the minimum information about a metagenome-assembled genome (MIMAG) standard, where high-quality MAGs met ≥90% completeness and ≤ 5% contamination and medium-quality MAGs met ≥50% completion and ≤ 10% contamination (Bowers et al., 2017). The first dataset is Genomes from Earth’s Microbiomes, a dataset assembled by the IGM/M Data Consortium from a variety of natural and engineered systems (Nayfach et al., 2021). Out of the entire collection of 52,515 medium and high-quality MAGs, MAGs with the metadata field “ecosystem_category” matching the query “wastewater” were selected for this analysis, resulting in a subset of 2,627 MAGs. We annotated the MAGs for coding regions and function using prokka v1.14.6 with default e-value threshold of 1e-06 (Seemann, 2014). The wastewater MAGs from this dataset were primarily represented by anaerobic digester samples, so a second set of MAGs representing activated sludge was also used and accessed through NCBI BioProject PRJNA629478. In this study, 1,083 high-quality MAGs were recovered through a combination of short-read and long-read sequencing (Singleton et al., 2021). In total, we analyzed 3,710 MAGs from activated sludge and wastewater bioreactors.

### 2.2. Activated sludge functional groups

Isolate genomes and high-quality MAGs from activated sludge functional groups were curated from NCBI. As a point of comparison, genomes of cyanobacteria and *Acinetobacter* with known cyanophycin metabolic pathways were also included. A complete list of genomes used is available in Supplementary Table S1. To understand patterns among genes clustered near *cphA*, conserved gene clusters with gene synteny 6,000 bp upstream and downstream of *cphA* were identified and binned using GeneGrouper, with search settings of > = 20% identity and > =80% coverage of the seed gene (McFarland et al., 2022).

### 2.3. Cyanophycin gene expression in phosphorus accumulating organisms

High-quality *Candidatus* ‘Accumulibacter’ (referred to herein as Accumulibacter) MAGs and associated metatranscriptomic data were used to examine *cphA* expression in Accumulibacter PAO. Our group previously operated a lab-scale denitrifying PAO reactor enriched in Accumulibacter (Gao et al., 2017; Wang et al., 2021). Three high quality *Ca.* Accumulibacter MAGs were assembled from this work belonging to clades IA, IC, and IF based on polyphosphate kinase (*ppk1*) gene phylogeny. MAGs and time-series metatranscriptomic data from this study were accessed through NCBI BioProject PRJNA576469. Raw RNA reads were filtered for quality with fastp (Chen et al., 2018) and reads mapping to rRNA were removed using BBMap against the SILVA database (Quast et al., 2012).^1^ Cleaned reads were aligned against Accumulibacter MAGs using kallisto (Bray et al., 2016). Expression levels of each mapped gene were normalized to Transcript per Million Reads (TPM). We also examined MAGs and expression levels in TPM from a lab-scale EBPR reactor where *Tetrasphaera* MAGs were recovered (McDaniel et al., 2022). Further operational details of the EBPR reactors can be found in their respective publications.

## 3. Results

### 3.1. Cyanophycin metabolism genes are widespread

**Figure 1:**
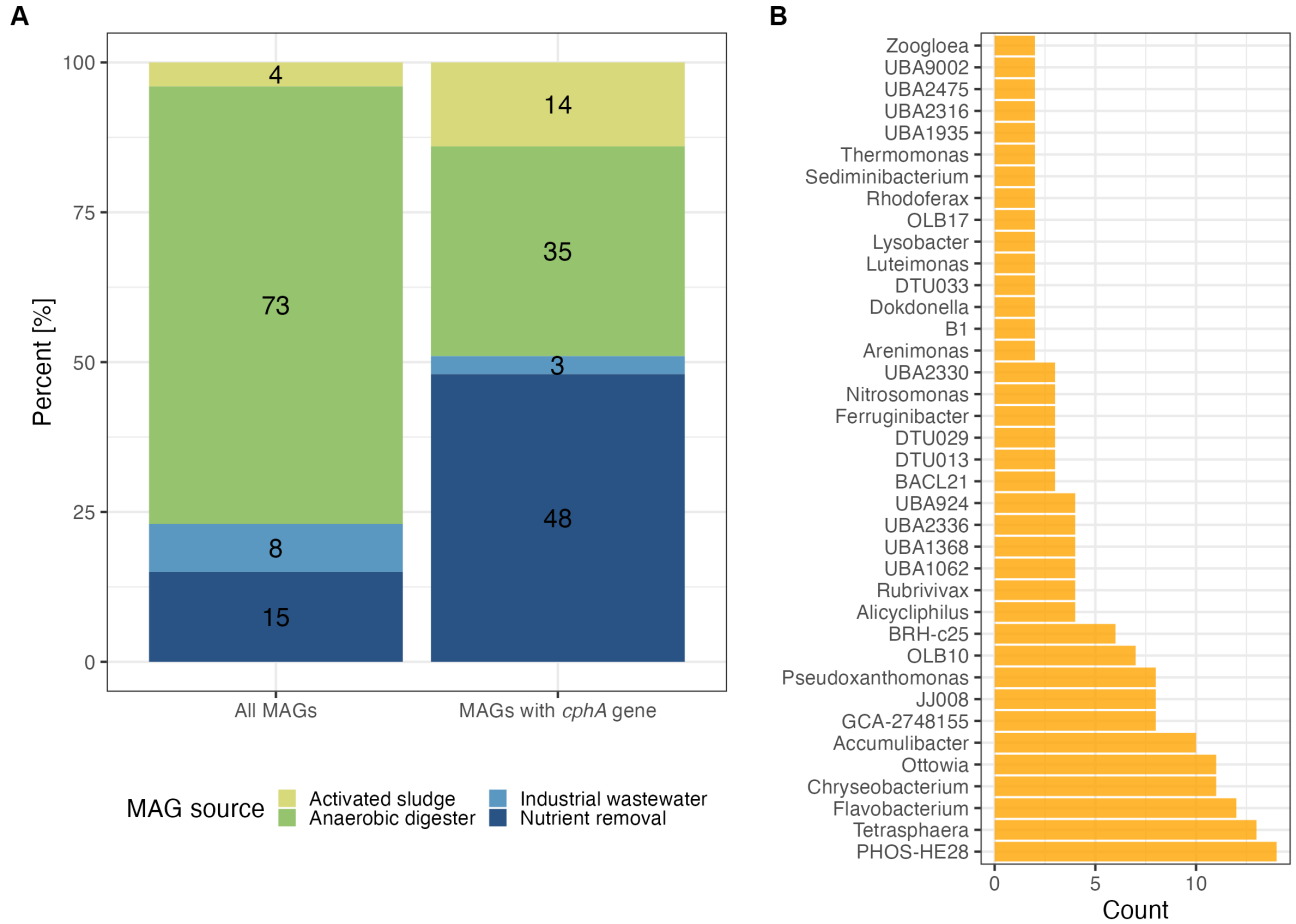
Distribution of MAGs from the Genomes from Earth’s Microbiomes collection (Nayfach et al., 2021) based on their identified ecosystem type with percent of total count labeled **(A)** and genus classifications of MAGs that had a *cphA* gene **(B)**. Data in panel **(B)** are shown for genus-level classifications of two or more MAGs based on GTDB taxonomy.

We searched for *cphA* in broad collections of wastewater-associated MAGs to understand the prevalence of the cyanophycin synthesis function. The first dataset we examined was primarily represented by MAGs recovered from anaerobic digester sludge (Figure 1). Around 10% of the MAGs, 271 out of 2,627, possessed the *cphA* gene. While nearly three quarters of the MAGs were derived from anaerobic digesters, only one third of these MAGs possessed a *cphA* gene (Figure 1A). On the other hand, the nutrient removal and activated sludge categories had greater proportions of MAGs with *cphA* compared to the original dataset. Examining the MAGs with *cphA* more closely, we found that key nutrient cycling organisms possessed a *cphA* gene, including known PAO Accumulibacter and *Tetrasphaera,* as well as ammonia oxidizing bacteria affiliated with the genus *Nitrosomonas* (Figure 1B). This result was surprising, because *cphA* had not been documented in *Ca.* Accumulibacter genomes to our knowledge.

A key limitation of the GEM dataset is the metadata categories. For example, separate nutrient removal and activated sludge categories are not representative of real wastewater treatment systems. Nutrient removal is often performed in activated sludge systems, where redox conditions are controlled to achieve N and P removal, such as the anaerobic-anoxic-oxic (A2O) process. One specific example from this dataset of potential category overlap is from Taxon Object ID 3300009540. This study was marked as activated sludge in the metadata, but further examination of the study (GOLD ID Gs0103597) shows that the samples were collected from an activated sludge system performing nitrification.

Given the metadata limitations of the GEM dataset, as well as the presence of *cphA* in nutrient cycling microbes, we next searched for *cphA* in a set of MAGs from activated sludge systems performing EBPR and N removal (Singleton et al., 2021). Out of 1,083 MAGs from this study, 552 possessed a *cphA* gene copy. Similar to the GEM dataset, we found that N and P cycling microbes harbored the *cphA* genes, including *Ca.* Accumulibacter, *Dechloromonas, Nitrosomonas*, and *Propionivibrio* (Supplementary Figure S2).

We also found that common filamentous bacteria possessed a *cphA* gene copy, including *Zoogloea* and *Kouleothrix.* Although filamentous bacteria are undesirable in large quantities in activated sludge systems due to their contribution to sludge bulking and poor settling, they are ubiquitous throughout activated sludge systems and can improve floc strength in balance with other microbes (Burger et al., 2017). The presence of *cphA* in filamentous bacteria may improve the viability of future cyanophycin applications, as filamentous bacteria can represent over 25% of sludge biomass in well-functioning systems (Mielczarek et al., 2012; Araújo Dos Santos et al., 2015).

A prominent *cphA* harboring genus in both large-scale datasets was PHOS-HE28. These organisms are poorly characterized members of the *Flavobacteriales* order. PHOS-HE28 have been identified in activated sludge and are related to bacteria isolated from saline environments, primarily seawater (Bowman, 2020). PHOS-HE28 and other related bacteria possess a *ppk1* gene, so it is possible that these bacteria can store phosphate like PAO (Lucena et al., 2022), though more examination of *ppk1* phylogeny and other phosphate transport genes is necessary to infer this function. Further investigation of PHOS-HE28 may be a promising avenue for integrating cyanophycin accumulation into existing treatment facilities given its ubiquity in activated sludge.

Overall, the unexpectedly high prevalence *cphA* genes in activated sludge MAGs is a positive sign that cyanophycin accumulation could integrate with existing wastewater treatment practices. This finding agrees with recent work that studied cyanophycin gene abundance and production in two full-scale wastewater treatment facilities using biofilm reactors for N and P removal (Zou et al., 2022). This work successfully identified *cphA* genes in biomass samples and found an association between *cphA* abundance and Accumulibacter marker gene abundance. However, they used read-based analysis for their metagenomic work rather than assembly-based analysis, while we analyzed MAGs to directly associate *cphA* genes with particular taxa. We next focused our analysis on specific N and P cycling organisms to better understand their potential for cyanophycin accumulation.

### 3.2. Nitrogen and phosphorus cycling bacteria harbor cyanophycin genes

Since *cphA* genes were widespread among wastewater treatment microbiomes, we next examined a wider suite of complete genomes and near-complete MAGs obtained from NCBI of N and P cycling microbes to determine their potential for cyanophycin accumulation. We selected genomes of ammonia oxidizing bacteria (AOB), nitrite oxidizing bacteria (NOB), denitrifiers, PAO, and GAO to search for the *cphA* gene. A complete list of genomes examined is available in Supplementary Table S1.

Out of 68 genomes searched, 34 possessed at least one copy of the *cphA* gene. Notably, nearly all PAO genomes possessed a copy of the *cphA* gene. All Accumulibacter and *Tetrasphaera* genomes had a copy, as well as *Ca.* ‘Dechloromonas phosphorivorans’. This finding is consistent with previous research; *cphA* has been identified in *Tetrasphaera* (Singleton et al., 2022), and correlations were found between Accumulibacter phylogenetic markers and *cphA* gene abundance (Zou et al., 2022). The only PAO genome without a *cphA* gene copy was one *Ca.* ‘Dechloromonas phosphorivorans’ genome. This result was surprising since the six other *Dechloromonas* species analyzed possessed a *cphA* gene. It is unclear whether this discrepancy is due to a true lack of *cphA* in this particular species or limitations in sequencing and metagenome assembly. Regardless, given that PAO are already harnessed for their affinity for P bioconcentration, the potential for simultaneous N recovery via synthesis of cyanophycin in the same organism may be a promising avenue for combined P and N recovery.

Another notable finding was that no NOB genome possessed a *cphA* gene, while multiple AOB genomes possessed a *cphA* gene copy. This finding was surprising since AOB and NOB are similar metabolically as chemolithoautotrophs. Furthermore, some *Nitrospira*-affiliated taxa previously thought to be NOB are capable of complete ammonia oxidation (comammox), resulting in even more metabolic similarities to AOB with the ability to oxidize ammonia (Daims et al., 2016). We analyzed a known comammox genome of *Ca.* Nitrospira nitrosa (van Kessel et al., 2015) and did not find a *cphA* gene. Among the AOB genera, we found *cphA* in all *Nitrosospira* genomes and seven out of 12 *Nitrosomonas* genomes. A previous study also identified *cphA* in a *Nitrosospira* originally isolated from soil (Norton et al., 2008). Of the five *Nitrosomonas* genomes that did not possess *cphA,* four were originally isolated from marine or brackish environments, not activated sludge, and required a salt-enriched medium for growth (Koops et al., 1991). The other *Nitrosomonas* genome that did not possess *cphA* was originally isolated from cattle manure (Nakagawa and Takahashi, 2015). The presence of *cphA* in common activated sludge AOB, such as *N. europaea* and *N. nitrosa*, is promising for future study and integration of cyanophycin accumulation with existing nitrification bioprocesses.

We also assessed the presence of *cphB* in all functional group genomes. Interestingly, out of all analyzed genomes, only *Tetrasphaera* species possessed a *cphB* gene. This was unlike the four cyanobacterial genomes, which all possessed a *cphB* gene copy. The lack of a *cphB* gene does not guarantee that an organism is incapable of depolymerizing cyanophycin, which has been confirmed in isolate cultures of *Pseudomonas aeruginosa* (Sharon et al., 2023). To better understand how cyanophycin could fit into a broader biosynthetic pathway, particularly in genomes lacking a *cphB* gene, we next examined the genes upstream and downstream of *cphA* in each genome.

### 3.3. Phosphorus accumulating organisms harbor distinct *cphA* gene clusters

We used GeneGrouper to identify genes surrounding *cphA* in each genome analyzed in section 3.2 and bin the gene clusters into homologous groups. We also included four cyanobacterial genomes and an *Acinetobacter* species with a well-characterized *cphA* gene to understand whether *cphA* gene clusters from the activated sludge functional groups were similar to known cyanophycin producers. This gene cluster grouping approach is useful for determining the potential role of cyanophycin in a broader bacterial metabolism; genes that cluster together in a genome often constitute a specific biosynthetic pathway or operon (Fischbach and Voigt, 2010), and operons have been shown to be conserved across bacterial classes (Brandis et al., 2019).

**Figure 2:**
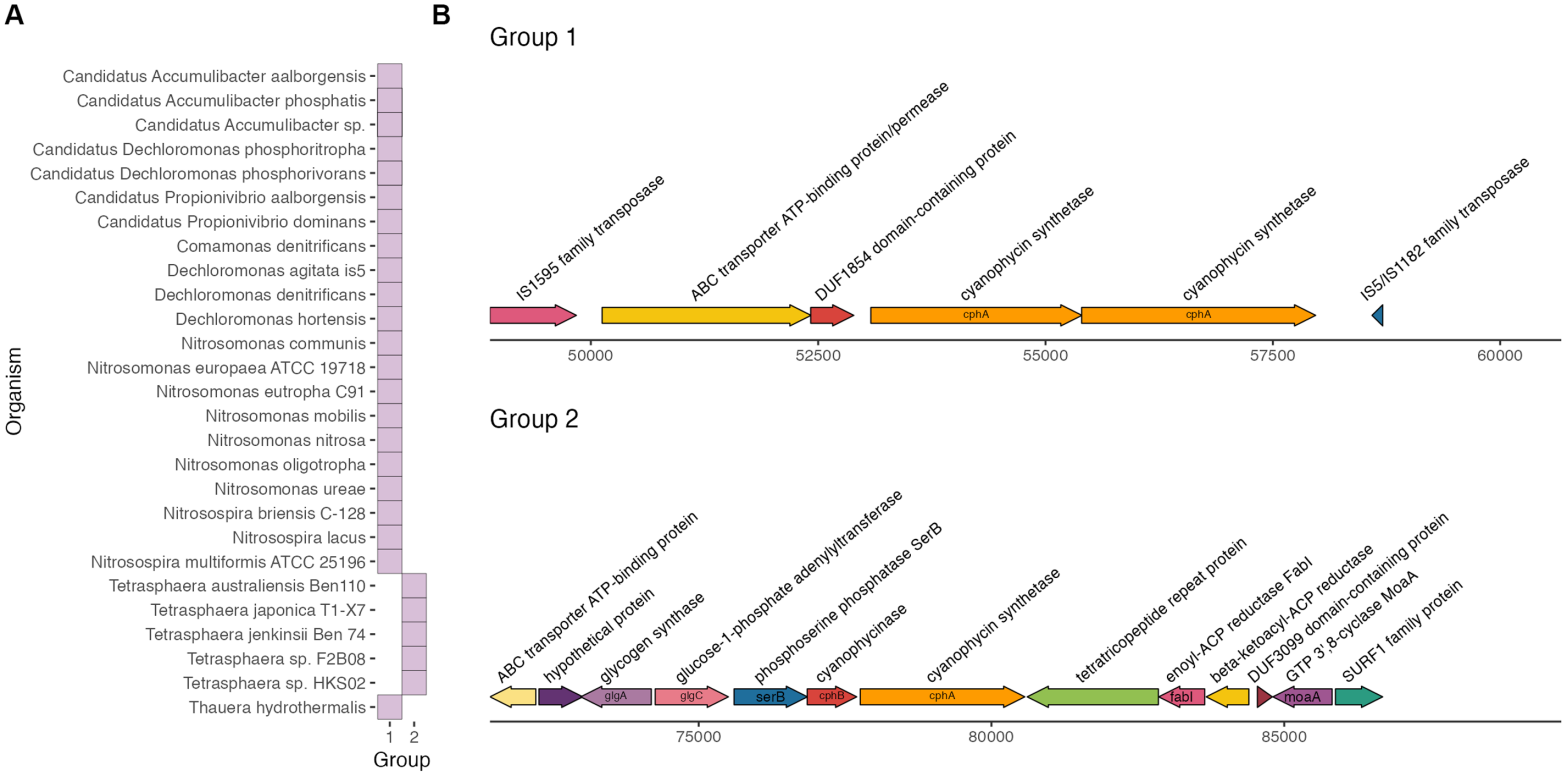
Groups of gene clusters **(A)** and gene cluster maps **(B)** around *cphA*.

We found two distinct gene clusters with gene synteny 6,000 bp upstream and downstream of *cphA,* shown in Figure 2. The *cphA* gene clusters from *Tetrasphaera* formed their own distinct group (Group 2), while the remainder of the genomes formed another group (Group 1). The reference cyanobacteria and *Acinetobacter* genomes did not form their own cluster nor cluster with the activated sludge taxa. The complexity of the Group 2 gene cluster was notable. The cluster consisted of a copy of *cphB* directly upstream of *cphA*, similar to *cphA* gene clusters of cyanobacteria (Krehenbrink et al., 2002; Füser and Steinbüchel, 2007). Furthermore, the Group 2 gene cluster harbored genes for glycogen synthesis and storage including glycogen synthetase *glgA* and glucose-1-adenylyltransferase *glgC,* as well as the amino acid utilization gene phosphoserine phosphatase *serB*. Glycogen is an important carbon reserve for *Tetrasphaera,* as they utilize glycogen under anaerobic conditions and replenish stores during aerobic conditions (Close et al., 2021; Yu et al., 2023).

The clustering of *cphA* and *cphB* with glycogen synthesis genes suggests that cyanophycin could be an active storage compound for *Tetrasphaera*. Genes in biosynthetic pathways with increasingly complex metabolites or related functions often cluster together, such as complex electron transport chains (Simon et al., 2004), protection against bacterial host immune response (Fischbach et al., 2006), and intracellular carbon storage (Kutralam-Muniasamy et al., 2017). Further analysis of carbon storage and amino acid utilization in *Tetrasphaera* can illuminate the role of cyanophycin as a storage compound.

The other *cphA* gene cluster group, Group 1, included a variety of activated sludge taxa. This gene cluster consisted of two copies of *cphA,* an unclassified transmembrane transport gene (ATP-binding ABC transporter), and two insertion sequences (IS). Notably, the gene cluster did not include a *cphB* gene copy. The presence of flanking IS indicates that this cluster could be a composite transposon, a type of mobile genetic element that facilitates movement of genetic material within a genome and between bacteria. Flanking IS around functional genes are a hallmark of composite transposons (Siguier et al., 2009). Composite transposons have been studied extensively for facilitating the spread of antibiotic resistance and xenobiotic resistance genes via horizontal gene transfer between taxa in diverse microbiomes (Top and Springael, 2003; Bennett, 2008). Mobile genetic elements have previously been identified as important vectors for horizontal gene transfer of antibiotic resistance genes in activated sludge microbiomes (Petrovich et al., 2018; Razavi et al., 2020), and recent work has also highlighted the role of composite transposons in transferring micropollutant degradation genes between bacteria (Bonatelli et al., 2023).

Further analysis of the *cphA* gene cluster in the Group 1 organisms would greatly improve our understanding of whether the gene cluster is a composite transposon or another type of mobile genetic element, which may have important implications for gene mobilization and transfer in complex microbial communities that are typical in wastewater bioprocesses. Future work could validate our findings through targeted PCR and long read sequencing to compare *cphA* gene clusters within activated sludge samples collected over time. Furthermore, the role of cyanophycin in the absence of *cphB* can be interrogated in future work that focuses on potential *cphB* analogs and cyanophycin transport genes.

### 3.4. Cyanophycin synthetase is expressed in phosphorus accumulating organisms

Since PAO would be an excellent candidate for combined N and P bioconcentration, we wanted to determine whether *cphA* could be expressed *in-situ* by these bacteria. We first examined gene expression by mapping metatranscriptomic reads against three high quality *Ca.* Accumulibacter MAGs assembled from a lab-scale denitrifying P removal bioreactor (Gao et al., 2019; Wang et al., 2021). The samples were obtained over complete reactor cycles consisting of three redox phases: anaerobic, anoxic (N supplied as nitrite), and aerobic. The reactor was fed with either acetate or propionate as a carbon source in equivalent concentrations on a COD basis. The three Accumulibacter MAGs affiliated with different clades (IA, IC, and IF) based on *ppk1* phylogeny and will be referred to hereafter by their clades. All three MAGs had two neighboring copies of *cphA* present in the genome. Neighboring copies of *cphA* have also been observed in other non-cyanobacterial genomes (Füser and Steinbüchel, 2007).

Each of the Accumulibacter MAGs exhibited different expression patterns across redox conditions; IA and IF had the greatest *cphA* expression during the aerobic phase, while IC had the greatest *cphA* expression during the anoxic phase (Supplementary Figure S3). Overall, IF had the greatest *cphA* expression, which agrees with previous findings that IF was the most transcriptionally active of all three MAGs (Wang et al., 2021). There were no apparent differences in *cphA* expression as a result of different carbon sources in the feed (Supplementary Figure S3).

**Figure 3:**
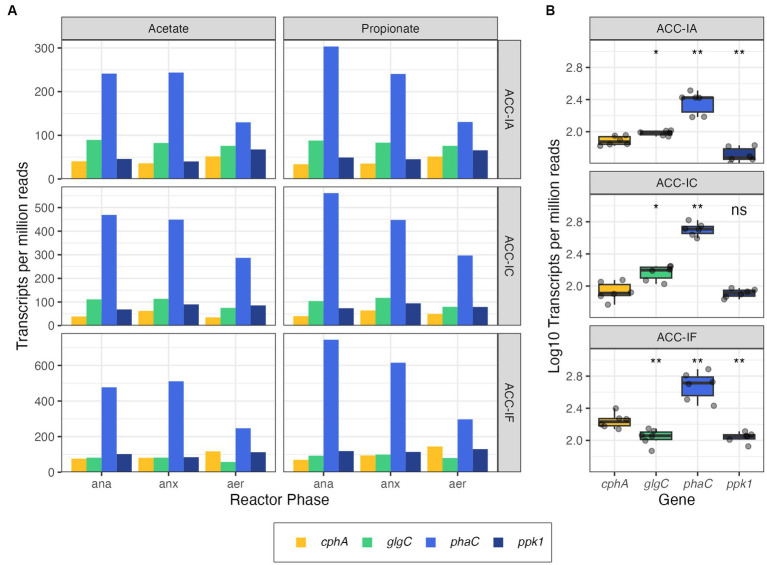
Gene expression profiles from metatranscriptomic reads of *cphA* (expression levels of both copies summed)*, glgC, phaC,* and *ppk1* in Accumulibacter MAGs plotted by the reactor phase (ana = anaerobic, anx = anoxic, aer = aerobic) and carbon source **(A)** and summarized per MAG **(B)**. Significance levels in panel **(B)** are based on the Wilcox rank sum test, where *glgC, phaC,* and *ppk* are compared against *cphA* in each subpanel (ns, not significant, *p < =0.05, **p < = 0.01).

In addition to analyzing the *cphA* expression of each MAG, we compared *cphA* expression to other key functional genes shown in Table 1. We used *ppk1, phaC,* and *glgC* as points of comparison against the expression of *cphA,* These genes are part of important phosphate transport and carbon storage functions and have been identified in a majority of *Ca.* Accumulibacter genomes (Petriglieri et al., 2022). The *ppk1* gene is an essential biomarker for PAO phosphate cycling activity and is expressed actively in EBPR processes (He et al., 2007; He and McMahon, 2011). Both *phaC* and *glgC* are used in carbon storage pathways. Accumulibacter PAO assimilate biodegradable substrate under anaerobic conditions and store the carbon intracellularly using polyhydroxyalkanoates (PHA), while simultaneously depleting glycogen reserves. Under anoxic or aerobic conditions, Accumulibacter replenish glycogen reserves while depleting PHA reserves (Lanham et al., 2014). As shown in Figure 3, *phaC* had the highest level of expression across all MAGs. Surprisingly, *cphA* expression was significantly higher than *ppk1* and *glgC* expression in IF. While gene expression does not directly indicate microbial activity or kinetics, the level of *cphA* expression relative to *ppk1* and *glgC* points to the possibility of a highly active cyanophycin synthesis pathway.

**Table 1:** Genes of interest for PAO expression analysis.

Abbreviation	Gene	Function
*cphA*	Cyanophycin synthetase	Cyanophycin polymerization
*glgC*	Glucose-1-phosphate adenylyltransferase	Glycogen polymerization
phaC^*^	Poly(3-hydroxyalkanoate) polymerase	PHA polymerization
*ppk1*	Polyphosphate kinase	Phosphate transport

*Not detected in *Tetrasphaera* MAGs.

We also examined gene expression data of two *Tetrasphaera* MAGs, TET1 and TET2, recovered from a time-series study of an EBPR bioreactor (McDaniel et al., 2022). These MAGs contained two copies of the *cphA* gene in series, similar to Accumulibacter and the Group 1 cluster from section 3.3. TET1 and TET2 had similar genes surrounding *cphA* as the other *Tetrasphaera* genomes examined in section 3.3, with a neighboring *cphB* as well as other carbon storage and cycling genes (Supplementary Figure S4). Furthermore, the *cphA* copies in TET1 and TET2 were not surrounded by flanking IS.

**Figure 4:**
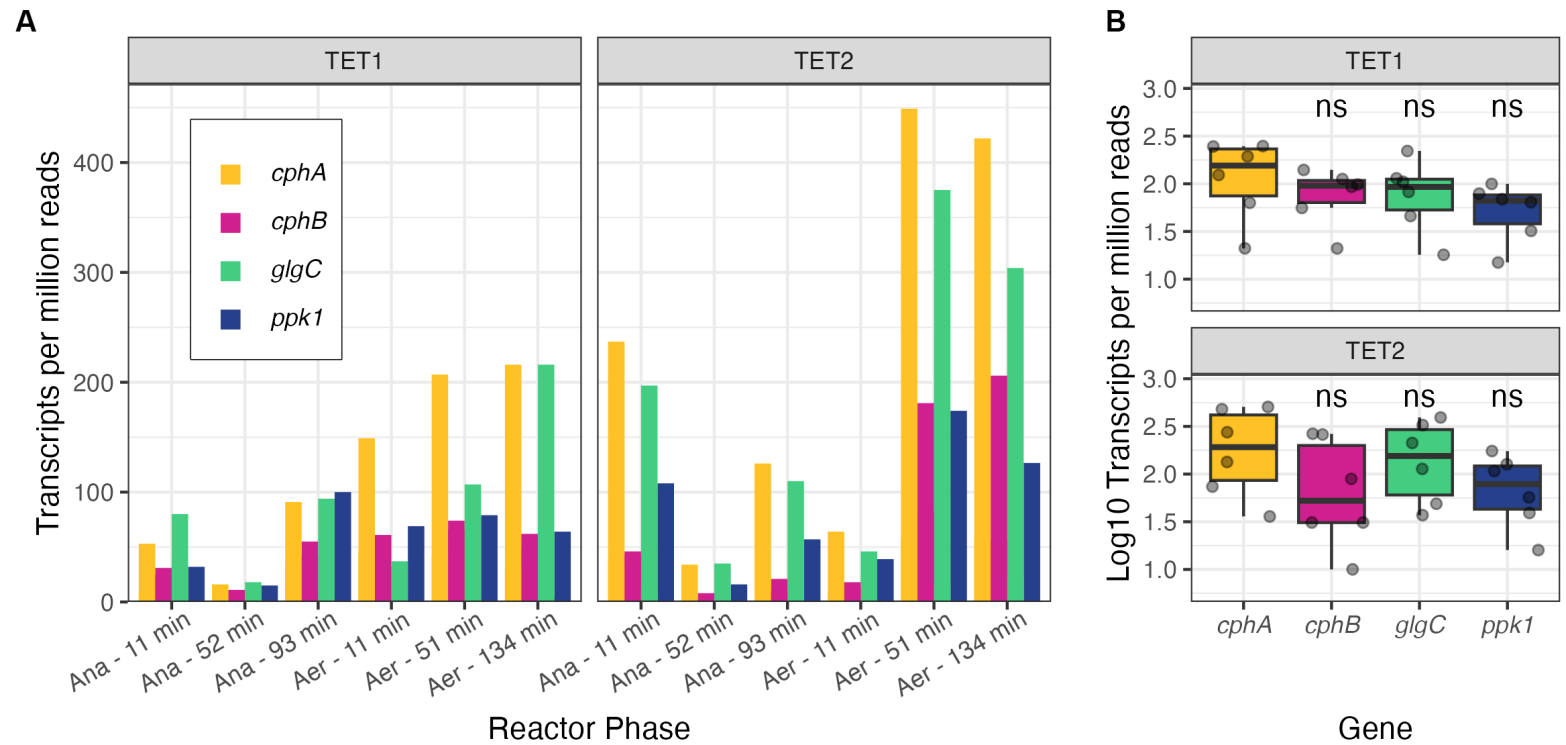
Gene expression profiles of *cphA* (expression levels of both copies summed), *cphB, glgC,* and *ppk1* in *Tetrasphaera* MAGs plotted by the reactor phase (Ana = anaerobic, Aer = aerobic) **(A)** and summarized per MAG **(B)** of TET1 and TET2 from McDaniel et al. (2022). Significance levels in panel **(B)** are based on the Wilcox rank sum test, where *cphB, glgC,* and *ppk1* are compared against *cphA* in each subpanel (ns, not significant).

Unlike the *cphA* expression in Accumulibacter, where both copies were expressed evenly, there was a notable difference in expression levels between the two *cphA* copies in the *Tetrasphaera* MAGs (Supplementary Figure S5). Notably, in both MAGs, the longer copy of *cphA* exhibited higher expression levels than the shorter copy; since the gene length is included in the normalization technique of calculating TPM, the gene length should not impact the reported expression levels. The combined gene expression of both *cphA* copies was relatively close to that of *glgC* and *ppk1* in the *Tetrasphaera* bins across the sampling period (Figure 4). Again, gene expression does not directly indicate degree of function or activity, but the comparable level of expression of *cphA* compared to well-understood genes of the PAO phenotype is a positive indication that cyanophycin is an active storage polymer for *Tetrasphaera*. We also observed gene expression of *cphB* in the *Tetrasphaera* MAGs (Figure 4), further increasing the likelihood that cyanophycin is actively produced and utilized in these bacteria.

The expression of *cphA* in PAO *Ca.* Accumulibacter and *Tetrasphaera* is promising for future applications of combined P and N accumulation. In particular, the location of the *cphA* gene in *Tetrasphaera* near other key carbon cycling genes, such *glgA* and *glgC* for glycogen synthesis, increases the likelihood that cyanophycin is an actively used biopolymer. Further analyses of cyanophycin pathway activity in response to operational variables and measurements of cyanophycin in real biomass would increase our confidence in successful simultaneous N and P bioconcentration in PAO.

## 4. Conclusion

In this study, we examined the prevalence of cyanophycin synthesis genes in wastewater bioprocess microbiomes. We observed a high prevalence of the *cphA* gene across a broad phylogenetic spectrum of common bacterial taxa in wastewater bioprocesses. The capacity for cyanophycin accumulation seems widespread given the presence of *cphA* in common PAO Accumulibacter, *Tetrasphaera,* and *Dechloromonas* and nitrifiers *Nitrosomonas* and *Nitrosospira.* We also used metatranscriptomic profiling to determine whether *cphA* genes were expressed by PAO under typical operating conditions, and found expression levels of *cphA* similar to other important P and carbon cycling genes. We also observed expression of *cphB* in *Tetrasphaera,* indicating that *Tetrasphaera* can actively produce and utilize cyanophycin. Overall, the presence of cyanophycin synthetase in nutrient cycling taxa suggests that cyanophycin cycling may already be occurring in existing biological nutrient removal processes.

Further research will expand on the findings of this work to advance N bioconcentration and fundamental microbial ecology questions. First, the feasibility of integrating cyanophycin accumulation into existing nutrient removal processes will largely depend on the ability to modulate cyanophycin production in concert with other desired functions, particularly P accumulation. Although we found that PAO harbor and express *cphA,* it is not clear whether cyanophycin accumulation occurs simultaneously with P accumulation. Second, fundamental understanding of cyanophycin accumulation by wastewater bioprocess taxa will improve with further examination of the *cphA* gene cluster as a possible mobile genetic element. While mobile genetic elements are intensely studied as a means of transferring antibiotic resistance genes in wastewater-associated microbiomes, their role in transferring nutrient cycling genes is less clear. Overall, our findings provide evidence that cyanophycin accumulation is a widespread function in nutrient removal bioprocesses and opens possibilities for accelerating nutrient recovery from wastewater through N bioconcentration.

## Data availability statement

The original contributions presented in the study are included in the article/Supplementary material, further inquiries can be directed to the corresponding author.

## Author contributions

MF: Conceptualization, Data curation, Formal analysis, Investigation, Methodology, Writing – original draft, Writing – review & editing. RR: Conceptualization, Data curation, Writing – review & editing. WT: Conceptualization, Funding acquisition, Writing – review & editing. KT: Conceptualization, Funding acquisition, Investigation, Writing – review & editing. GW: Conceptualization, Funding acquisition, Supervision, Writing – review & editing.

## Footnotes

^1^ sourceforge.net/projects/bbmap/

## References

[ref1] Araújo Dos Santos L. Ferreira V. Neto M. M. Pereira M. A. Mota M. Nicolau A. (2015). Study of 16 Portuguese activated sludge systems based on filamentous bacteria populations and their relationships with environmental parameters. Appl. Microbiol. Biotechnol. 99, 5307–5316. doi: 10.1007/s00253-015-6393-8 25666680

[ref2] Batstone D. J. Hülsen T. Mehta C. M. Keller J. (2015). Platforms for energy and nutrient recovery from domestic wastewater: a review. Chemosphere 140, 2–11. doi: 10.1016/j.chemosphere.2014.10.021 25455679

[ref3] Beckinghausen A. Odlare M. Thorin E. Schwede S. (2020). From removal to recovery: an evaluation of nitrogen recovery techniques from wastewater. Appl. Energy 263:114616. doi: 10.1016/j.apenergy.2020.114616

[ref4] Bennett P. M. (2008). Plasmid encoded antibiotic resistance: acquisition and transfer of antibiotic resistance genes in bacteria: plasmid-encoded antibiotic resistance. Br. J. Pharmacol. 153, S347–S357. doi: 10.1038/sj.bjp.0707607 18193080 PMC2268074

[ref5] Berg H. Ziegler K. Piotukh K. Baier K. Lockau W. Volkmer-Engert R. (2000). Biosynthesis of the cyanobacterial reserve polymer multi-L-arginyl-poly-L-aspartic acid (cyanophycin): mechanism of the cyanophycin synthetase reaction studied with synthetic primers. Eur. J. Biochem. 267, 5561–5570. doi: 10.1046/j.1432-1327.2000.01622.x 10951215

[ref6] Bonatelli M. L. Rohwerder T. Popp D. Liu Y. Akay C. Schultz C.. (2023). Recently evolved combination of unique sulfatase and amidase genes enables bacterial degradation of the wastewater micropollutant acesulfame worldwide. Front. Microbiol. 14:1223838. doi: 10.3389/fmicb.2023.1223838 37577448 PMC10413263

[ref7] Bowers R. M. Kyrpides N. C. Stepanauskas R. Harmon-Smith M. Doud D. Reddy T. B. K.. (2017). Minimum information about a single amplified genome (MISAG) and a metagenome-assembled genome (MIMAG) of bacteria and archaea. Nat. Biotechnol. 35, 725–731. doi: 10.1038/nbt.3893 28787424 PMC6436528

[ref8] Bowman J. P. (2020). Out from the shadows – resolution of the taxonomy of the family cryomorphaceae. Front. Microbiol. 11:795. doi: 10.3389/fmicb.2020.00795 32431677 PMC7214798

[ref9] Brandis G. Cao S. Hughes D. (2019). Operon concatenation is an ancient feature that restricts the potential to rearrange bacterial chromosomes. Mol. Biol. Evol. 36, 1990–2000. doi: 10.1093/molbev/msz129 31132113 PMC6735719

[ref10] Bray N. L. Pimentel H. Melsted P. Pachter L. (2016). Near-optimal probabilistic RNA-seq quantification. Nat. Biotechnol. 34, 525–527. doi: 10.1038/nbt.3519 27043002

[ref11] Burger W. Krysiak-Baltyn K. Scales P. J. Martin G. J. O. Stickland A. D. Gras S. L. (2017). The influence of protruding filamentous bacteria on floc stability and solid-liquid separation in the activated sludge process. Water Res. 123, 578–585. doi: 10.1016/j.watres.2017.06.063 28704773

[ref12] Chen S. Zhou Y. Chen Y. Gu J. (2018). fastp: an ultra-fast all-in-one FASTQ preprocessor. Bioinformatics 34, i884–i890. doi: 10.1093/bioinformatics/bty560 30423086 PMC6129281

[ref13] Close K. Marques R. Carvalho V. C. F. Freitas E. B. Reis M. A. M. Carvalho G.. (2021). The storage compounds associated with *Tetrasphaera* PAO metabolism and the relationship between diversity and P removal. Water Res. 204:117621. doi: 10.1016/j.watres.2021.117621 34500182

[ref14] Daims H. Lücker S. Wagner M. (2016). A new perspective on microbes formerly known as nitrite-oxidizing bacteria. Trends Microbiol. 24, 699–712. doi: 10.1016/j.tim.2016.05.004 27283264 PMC6884419

[ref15] Du J. Li L. Zhou S. (2019). Microbial production of cyanophycin: from enzymes to biopolymers. Biotechnol. Adv. 37:107400. doi: 10.1016/j.biotechadv.2019.05.006 31095967

[ref16] Elbahloul Y. Krehenbrink M. Reichelt R. Steinbüchel A. (2005). Physiological conditions conducive to high cyanophycin content in biomass of *Acinetobacter calcoaceticus* strain ADP1. Appl. Environ. Microbiol. 71, 858–866. doi: 10.1128/AEM.71.2.858-866.2005 15691941 PMC546767

[ref17] Fischbach M. A. Lin H. Zhou L. Yu Y. Abergel R. J. Liu D. R.. (2006). The pathogen-associated *iroA* gene cluster mediates bacterial evasion of lipocalin 2. Proc. Natl. Acad. Sci. 103, 16502–16507. doi: 10.1073/pnas.0604636103 17060628 PMC1637611

[ref18] Fischbach M. Voigt C. A. (2010). Prokaryotic gene clusters: a rich toolbox for synthetic biology. Biotechnol. J. 5, 1277–1296. doi: 10.1002/biot.201000181 21154668 PMC3904232

[ref19] Flores E. Arévalo S. Burnat M. (2019). Cyanophycin and arginine metabolism in cyanobacteria. Algal Res. 42:101577. doi: 10.1016/j.algal.2019.101577

[ref20] Füser G. Steinbüchel A. (2005). Investigations on the solubility behavior of cyanophycin. Solubility of cyanophycin in solutions of simple inorganic salts. Biomacromolecules 6, 1367–1374. doi: 10.1021/bm049371o 15877354

[ref21] Füser G. Steinbüchel A. (2007). Analysis of genome sequences for genes of cyanophycin metabolism: identifying putative cyanophycin metabolizing prokaryotes. Macromol. Biosci. 7, 278–296. doi: 10.1002/mabi.200600207 17390395

[ref22] Galloway J. N. Townsend A. R. Erisman J. W. Bekunda M. Cai Z. Freney J. R.. (2008). Transformation of the nitrogen cycle: recent trends, questions, and potential solutions. Science 320, 889–892. doi: 10.1126/science.1136674 18487183

[ref23] Gao H. Liu M. Griffin J. S. Xu L. Xiang D. Scherson Y. D.. (2017). Complete nutrient removal coupled to nitrous oxide production as a bioenergy source by denitrifying polyphosphate-accumulating organisms. Environ. Sci. Technol. 51, 4531–4540. doi: 10.1021/acs.est.6b04896 28212019

[ref24] Gao H. Mao Y. Zhao X. Liu W.-T. Zhang T. Wells G. (2019). Genome-centric metagenomics resolves microbial diversity and prevalent truncated denitrification pathways in a denitrifying PAO-enriched bioprocess. Water Res. 155, 275–287. doi: 10.1016/j.watres.2019.02.020 30852315

[ref25] He S. Gall D. L. McMahon K. D. (2007). “*Candidatus* Accumulibacter” population structure in enhanced biological phosphorus removal sludges as revealed by polyphosphate kinase genes. Appl. Environ. Microbiol. 73, 5865–5874. doi: 10.1128/AEM.01207-07 17675445 PMC2074919

[ref26] He S. McMahon K. D. (2011). ‘*Candidatus* Accumulibacter’ gene expression in response to dynamic EBPR conditions. ISME J. 5, 329–340. doi: 10.1038/ismej.2010.127 20703317 PMC3105699

[ref27] Koops H. P. Böher B. Mör U. C. Pommerening-Röser A. Stehr G. (1991). Classification of eight new species of ammonia-oxidizing bacteria: *Nitrosomonas communis* sp. nov., *Nitrosomonas ureae* sp. nov., *Nitrosomonas aestuarii* sp. nov., *Nitrosomonas marina* sp. nov., *Nitrosomonas nitrosa* sp. nov., *Nitrosomonas eutropha* sp. nov., *Nitrosomonas oligotropha* sp. nov. and *Nitrosomonas halophila* sp. nov. Microbiology 137, 1689–1699. doi: 10.1099/00221287-137-7-1689

[ref28] Krehenbrink M. Oppermann-Sanio F.-B. Steinbüchel A. (2002). Evaluation of non-cyanobacterial genome sequences for occurrence of genes encoding proteins homologous to cyanophycin synthetase and cloning of an active cyanophycin synthetase from *Acinetobacter* sp. strain DSM 587. Arch. Microbiol. 177, 371–380. doi: 10.1007/s00203-001-0396-9 11976746

[ref29] Kutralam-Muniasamy G. Corona-Hernandez J. Narayanasamy R.-K. Marsch R. Pérez-Guevara F. (2017). Phylogenetic diversification and developmental implications of poly-(R)-3-hydroxyalkanoate gene cluster assembly in prokaryotes. FEMS Microbiol. Lett. 364:135. doi: 10.1093/femsle/fnx135 28655209

[ref30] Lanham A. B. Oehmen A. Saunders A. M. Carvalho G. Nielsen P. H. Reis M. A. M. (2014). Metabolic modelling of full-scale enhanced biological phosphorus removal sludge. Water Res. 66, 283–295. doi: 10.1016/j.watres.2014.08.036 25222332

[ref31] Lucena T. Sánchez O. Sanz-Saez I. Acinas S. G. Garrido L. Mas J.. (2022). *Parvicella tangerina* gen. nov., sp. nov. (*Parvicellaceae* fam. nov., *Flavobacteriales*), first cultured representative of the marine clade UBA10066, and *Lysobacter luteus* sp. nov., from activated sludge of a seawater-processing wastewater treatment plant. Int. J. Syst. Evol. Microbiol. 72:5498. doi: 10.1099/ijsem.0.005498 35997078

[ref32] McDaniel E. A. Van Steenbrugge J. J. M. Noguera D. R. McMahon K. D. Raaijmakers J. M. Medema M. H.. (2022). TbasCO: trait-based comparative ‘omics identifies ecosystem-level and niche-differentiating adaptations of an engineered microbiome. ISME Commun. 2:111. doi: 10.1038/s43705-022-00189-2 37938301 PMC9723799

[ref33] McFarland A. G. Kennedy N. W. Mills C. E. Tullman-Ercek D. Huttenhower C. Hartmann E. M. (2022). Density-based binning of gene clusters to infer function or evolutionary history using GeneGrouper. Bioinformatics 38, 612–620. doi: 10.1093/bioinformatics/btab752 34734968

[ref34] Mielczarek A. T. Kragelund C. Eriksen P. S. Nielsen P. H. (2012). Population dynamics of filamentous bacteria in Danish wastewater treatment plants with nutrient removal. Water Res. 46, 3781–3795. doi: 10.1016/j.watres.2012.04.009 22608099

[ref35] Nakagawa T. Takahashi R. (2015). *Nitrosomonas stercoris* sp. nov., a chemoautotrophic ammonia-oxidizing bacterium tolerant of high ammonium isolated from composted cattle manure. Microbes Environ. 30, 221–227. doi: 10.1264/jsme2.ME15072 26156554 PMC4567560

[ref36] Nausch H. Dorn M. Frolov A. Hoedtke S. Wolf P. Broer I. (2020). Direct delivery of health promoting β-asp-arg dipeptides via stable co-expression of cyanophycin and the cyanophycinase CphE241 in tobacco plants. Front. Plant Sci. 11:842. doi: 10.3389/fpls.2020.00842 32636862 PMC7318851

[ref37] Nausch H. Hausmann T. Ponndorf D. Hühns M. Hoedtke S. Wolf P.. (2016). Tobacco as platform for a commercial production of cyanophycin. New Biotechnol. 33, 842–851. doi: 10.1016/j.nbt.2016.08.001 27501906

[ref38] Nayfach S. Roux S. Seshadri R. Udwary D. Varghese N. Schulz F.. (2021). A genomic catalog of earth’s microbiomes. Nat. Biotechnol. 39, 499–509. doi: 10.1038/s41587-020-0718-6 33169036 PMC8041624

[ref39] Neumann K. Stephan D. P. Ziegler K. Hühns M. Broer I. Lockau W.. (2005). Production of cyanophycin, a suitable source for the biodegradable polymer polyaspartate, in transgenic plants: production of cyanophycin in transgenic plants. Plant Biotechnol. J. 3, 249–258. doi: 10.1111/j.1467-7652.2005.00122.x 17173624

[ref40] Norton J. M. Klotz M. G. Stein L. Y. Arp D. J. Bottomley P. J. Chain P. S. G.. (2008). Complete genome sequence of *Nitrosospira multiformis*, an ammonia-oxidizing bacterium from the soil environment. Appl. Environ. Microbiol. 74, 3559–3572. doi: 10.1128/AEM.02722-07 18390676 PMC2423025

[ref41] Petriglieri F. Singleton C. M. Kondrotaite Z. Dueholm M. K. D. McDaniel E. A. McMahon K. D.. (2022). Reevaluation of the phylogenetic diversity and global distribution of the genus “*Candidatus* Accumulibacter.”. mSystems 7:e0001622. doi: 10.1128/msystems.00016-22 35467400 PMC9238405

[ref42] Petrovich M. Chu B. Wright D. Griffin J. Elfeki M. Murphy B. T.. (2018). Antibiotic resistance genes show enhanced mobilization through suspended growth and biofilm-based wastewater treatment processes. FEMS Microbiol. Ecol. 94:fiy041. doi: 10.1093/femsec/fiy041 29534199

[ref43] Quast C. Pruesse E. Yilmaz P. Gerken J. Schweer T. Yarza P.. (2012). The SILVA ribosomal RNA gene database project: improved data processing and web-based tools. Nucleic Acids Res. 41, D590–D596. doi: 10.1093/nar/gks1219 23193283 PMC3531112

[ref44] Razavi M. Kristiansson E. Flach C.-F. Larsson D. G. J. (2020). The association between insertion sequences and antibiotic resistance genes. mSphere 5:e00418-20. doi: 10.1128/mSphere.00418-20 32878926 PMC7471000

[ref45] Richter R. Hejazi M. Kraft R. Ziegler K. Lockau W. (1999). Cyanophycinase, a peptidase degrading the cyanobacterial reserve material multi-L-arginyl-poly-L-aspartic acid (cyanophycin). Molecular cloning of the gene of *Synechocystis* sp. PCC 6803, expression in *Escherichia coli*, and biochemical characterization of the purified enzyme. Eur. J. Biochem. 263, 163–169. doi: 10.1046/j.1432-1327.1999.00479.x 10429200

[ref46] Seemann T. (2014). Prokka: rapid prokaryotic genome annotation. Bioinformatics 30, 2068–2069. doi: 10.1093/bioinformatics/btu153 24642063

[ref47] Sharon I. McKay G. A. Nguyen D. Schmeing T. M. (2023). Discovery of cyanophycin dipeptide hydrolase enzymes suggests widespread utility of the natural biopolymer cyanophycin. Proc. Natl. Acad. Sci. 120:e2216547120. doi: 10.1073/pnas.2216547120 36800389 PMC9974463

[ref48] Siguier P. Gagnevin L. Chandler M. (2009). The new IS1595 family, its relation to IS1 and the frontier between insertion sequences and transposons. Res. Microbiol. 160, 232–241. doi: 10.1016/j.resmic.2009.02.003 19286454

[ref49] Simon J. Einsle O. Kroneck P. M. H. Zumft W. G. (2004). The unprecedented *nos* gene cluster of *Wolinella succinogenes* encodes a novel respiratory electron transfer pathway to cytochrome *c* nitrous oxide reductase. FEBS Lett. 569, 7–12. doi: 10.1016/j.febslet.2004.05.060 15225600

[ref50] Singleton C. M. Petriglieri F. Kristensen J. M. Kirkegaard R. H. Michaelsen T. Y. Andersen M. H.. (2021). Connecting structure to function with the recovery of over 1000 high-quality metagenome-assembled genomes from activated sludge using long-read sequencing. Nat. Commun. 12:2009. doi: 10.1038/s41467-021-22203-2 33790294 PMC8012365

[ref51] Singleton C. M. Petriglieri F. Wasmund K. Nierychlo M. Kondrotaite Z. Petersen J. F.. (2022). The novel genus, ‘*Candidatus* Phosphoribacter’, previously identified as Tetrasphaera, is the dominant polyphosphate accumulating lineage in EBPR wastewater treatment plants worldwide. ISME J. 16, 1605–1616. doi: 10.1038/s41396-022-01212-z 35217776 PMC9123174

[ref52] Top E. M. Springael D. (2003). The role of mobile genetic elements in bacterial adaptation to xenobiotic organic compounds. Curr. Opin. Biotechnol. 14, 262–269. doi: 10.1016/S0958-1669(03)00066-1 12849778

[ref53] Uddin Z. Fang T. Siao J. Tseng W. (2020). Wound healing attributes of polyelectrolyte multilayers prepared with multi- l -arginyl-poly- l -aspartate pairing with hyaluronic acid and γ-polyglutamic acid. Macromol. Biosci. 20:2000132. doi: 10.1002/mabi.202000132 32567226

[ref54] van Kessel M. A. H. Speth D. R. Albertsen M. Nielsen P. H. Camp H. J. M. O. Kartal B.. (2015). Complete nitrification by a single microorganism. Nature 528:555+. doi: 10.1038/nature16459 26610025 PMC4878690

[ref55] Wang Y. Gao H. Wells G. (2021). Integrated omics analyses reveal differential gene expression and potential for cooperation between denitrifying polyphosphate and glycogen accumulating organisms. Environ. Microbiol. 23, 3274–3293. doi: 10.1111/1462-2920.15486 33769674

[ref56] Yu Z. Xu S. Wang P. Liu D. Lu H. (2023). Phosphorus removal and storage polymer synthesis by *Tetrasphaera* -related bacteria with different carbon sources. ACS EST Water 3, 1243–1421. doi: 10.1021/acsestwater.3c00046

[ref57] Ziegler K. Diener A. Herpin C. Richter R. Deutzmann R. Lockau W. (1998). Molecular characterization of cyanophycin synthetase, the enzyme catalyzing the biosynthesis of the cyanobacterial reserve material multi- L-arginyl-poly- L-aspartate (cyanophycin). Eur. J. Biochem. 254, 154–159. doi: 10.1046/j.1432-1327.1998.2540154.x 9652408

[ref58] Zou K. Huang Y. Feng B. Qing T. Zhang P. Chen Y.-P. (2022). Cyanophycin granule polypeptide: a neglected high value-added biopolymer, synthesized in activated sludge on a large scale. Appl. Environ. Microbiol. 88:e0074222. doi: 10.1128/aem.00742-22 35862662 PMC9317870

