# Supplementary material for: Meta-omic profiling reveals ubiquity of genes encoding for the nitrogen-rich biopolymer cyanophycin in activated sludge microbiomes
Source: Front Microbiol. 2023 Nov 16;14:1287491. doi: 10.3389/fmicb.2023.1287491 (PMC10687191; doi:10.3389/fmicb.2023.1287491)
Supplement: Supplementary file 2 [file Data_Sheet_1.docx]

Supplementary Material


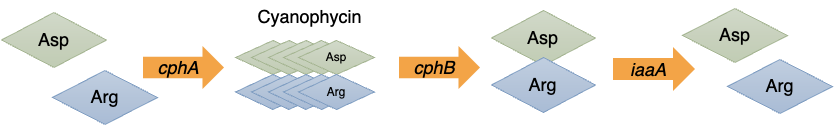
Figure S1. Cyanophycin metabolic pathway.


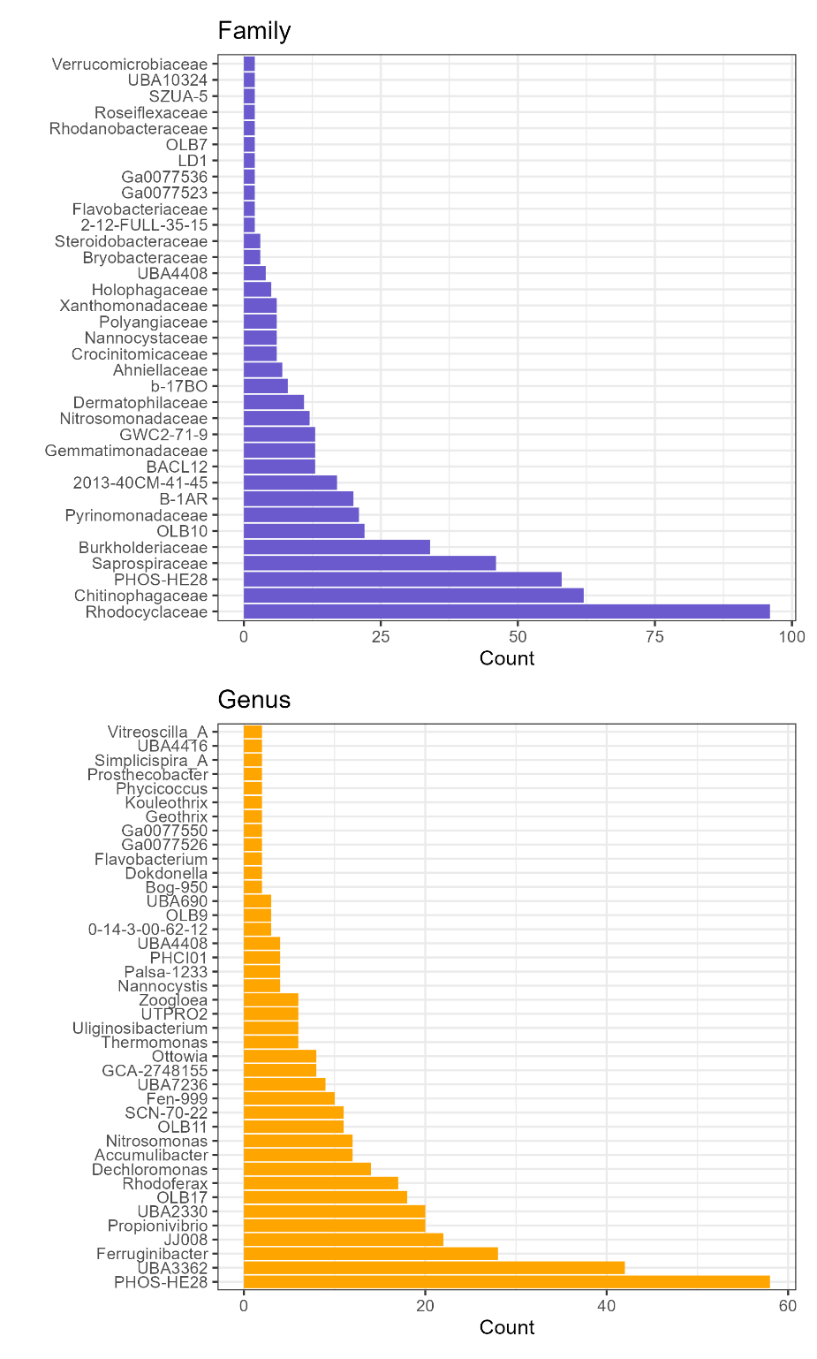


Figure S2. Family (top) and genus (bottom) classifications of MAGs from the Singleton et al., 2021 dataset that contained a *cphA* gene. Data shown in this figure are for family or genus classifications of two or more MAGs. Unknown taxonomic classifications are not shown.


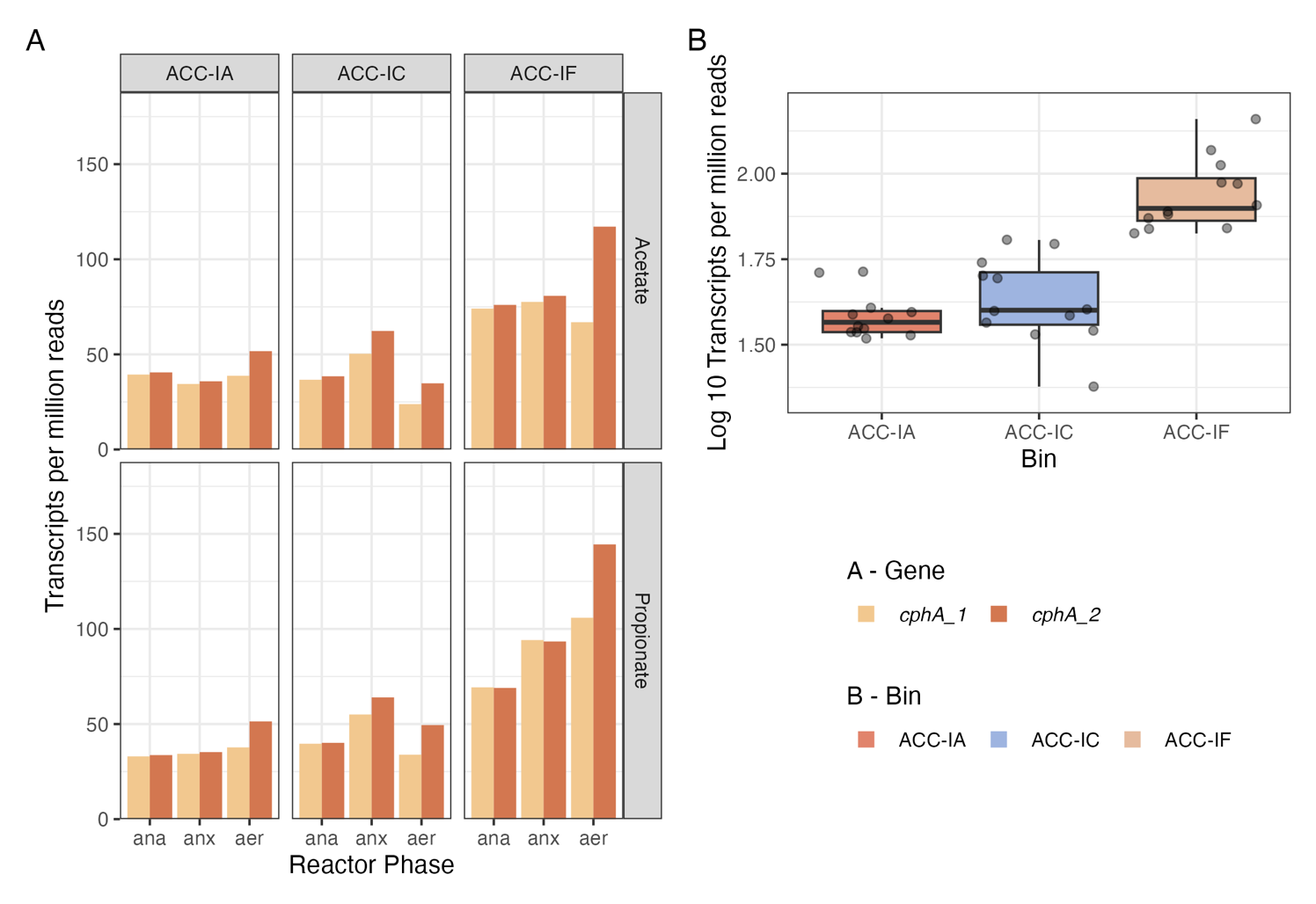


Figure S3. Gene expression profiles of *cphA* in *Ca.* Accumulibacter MAGs from Gao et al., 2019 and Wang et al., 2021 by *cphA* gene copy, reactor phase and carbon source (A) and both gene copies summarized by bin (B).


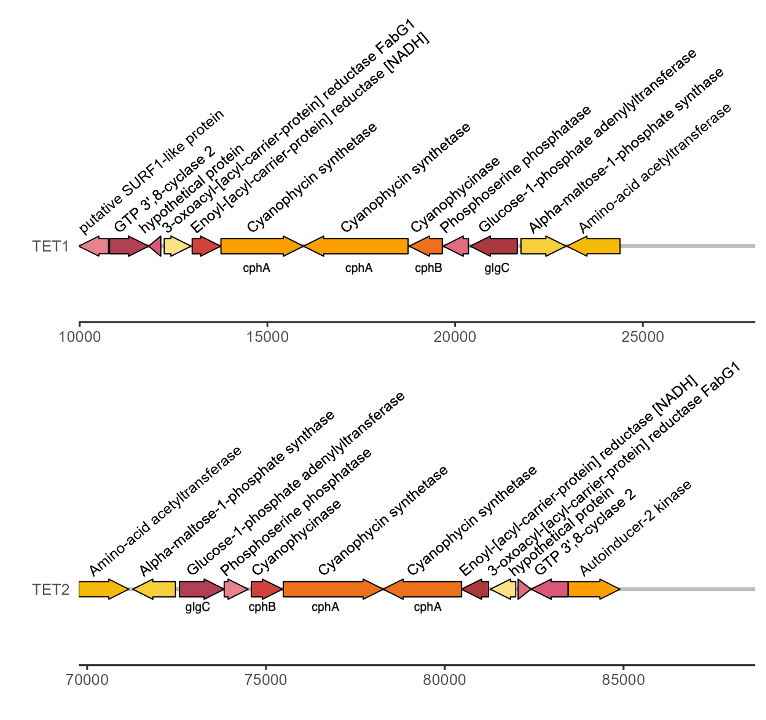


Figure S4. Gene map of TET1 and TET2 from McDaniel et al., 2022 around the *cphA* gene.


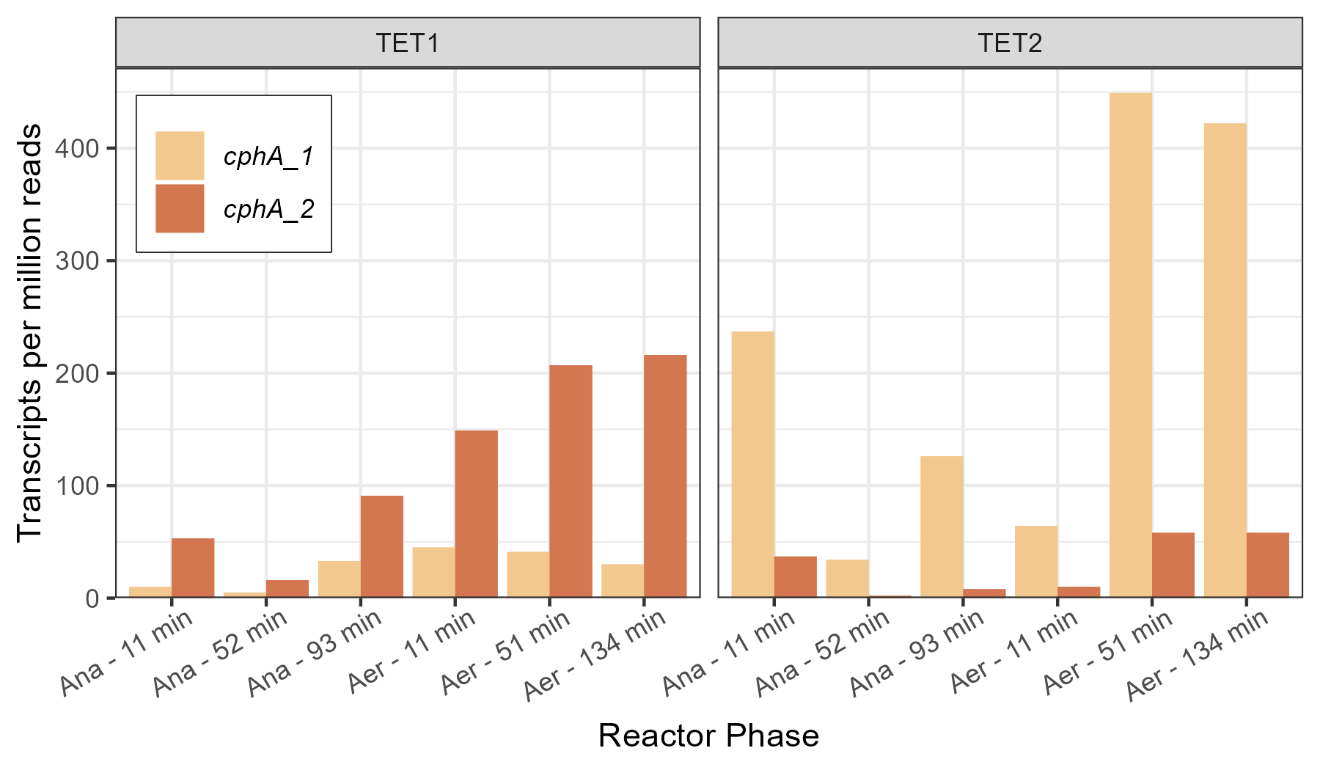


Figure S5. Gene expression profiles of *cphA* in *Tetrasphaera* MAGs from McDaniel et al. 2022 by bin and *cphA* gene copy. Ana = anaerobic and Aer = aerobic.

**References**

Gao, H., Mao, Y., Zhao, X., Liu, W.-T., Zhang, T., Wells, G., 2019. Genome-centric metagenomics resolves microbial diversity and prevalent truncated denitrification pathways in a denitrifying PAO-enriched bioprocess. Water Res. 155, 275–287. https://doi.org/10.1016/j.watres.2019.02.020

McDaniel, E.A., Van Steenbrugge, J.J.M., Noguera, D.R., McMahon, K.D., Raaijmakers, J.M., Medema, M.H., Oyserman, B.O., 2022. TbasCO: trait-based comparative ‘omics identifies ecosystem-level and niche-differentiating adaptations of an engineered microbiome. ISME Commun. 2, 111. https://doi.org/10.1038/s43705-022-00189-2

Singleton, C.M., Petriglieri, F., Kristensen, J.M., Kirkegaard, R.H., Michaelsen, T.Y., Andersen, M.H., Kondrotaite, Z., Karst, S.M., Dueholm, M.S., Nielsen, P.H., Albertsen, M., 2021. Connecting structure to function with the recovery of over 1000 high-quality metagenome-assembled genomes from activated sludge using long-read sequencing. Nat. Commun. 12, 2009. https://doi.org/10.1038/s41467-021-22203-2

Wang, Y., Gao, H., Wells, G., 2021. Integrated omics analyses reveal differential gene expression and potential for cooperation between denitrifying polyphosphate and glycogen accumulating organisms. Environ. Microbiol. 23, 3274–3293. https://doi.org/10.1111/1462-2920.15486
